# Emergency cricothyrotomy – a systematic review

**DOI:** 10.1186/1757-7241-21-43

**Published:** 2013-05-31

**Authors:** Sofie Langvad, Per Kristian Hyldmo, Anders Rostrup Nakstad, Gunn Elisabeth Vist, Marten Sandberg

**Affiliations:** 1 Faculty of Medicine, University of Oslo, Oslo, Norway; 2 Department of Research, Norwegian Air Ambulance Foundation, Drøbak, Norway; 3 Faculty of Social Sciences, University of Stavanger, Stavanger, Norway; 4 Air Ambulance Department, Oslo University Hospital, Sykehusveien 19, N-1474, Nordbyhagen, Norway; 5 Norwegian Knowledge Centre for the Health Services, PO Box 7004St Olavs Plass, N-0130, Oslo, Norway

**Keywords:** Emergency, Airway management, Cricothyrotomy, Surgical airway, Cannot intubate cannot ventilate

## Abstract

**Background:**

An emergency cricothyrotomy is the last-resort in most airway management protocols and is performed when it is not possible to intubate or ventilate a patient. This situation can rapidly prove fatal, making it important to identify the best method to establish a secure airway. We conducted a systematic review to identify whether there exists superiority between available commercial kits versus traditional surgical and needle techniques.

**Methods:**

Medline, EMBASE and other databases were searched for pertinent studies. The inclusion criteria included manikin, animal and human studies and there were no restrictions regarding the professional background of the person performing the procedure.

**Results:**

In total, 1,405 unique references were identified; 108 full text articles were retrieved; and 24 studies were included in the review. Studies comparing kits with one another or with various surgical and needle techniques were identified. The outcome measures included in this systematic review were success rate and time consumption. The investigators performing the studies had chosen unique combinations of starting and stopping points for time measurements, making comparisons between studies difficult and leading to many conflicting results. No single method was shown to be better than the others, but the size of the studies makes it impossible to draw firm conclusions.

**Conclusions:**

The large majority of the studies were too small to demonstrate statistically significant differences, and the limited available evidence was of low or very low quality. That none of the techniques in these studies demonstrated better results than the others does not necessarily indicate that each is equally good, and these conclusions will likely change as new evidence becomes available.

## Background

Emergency cricothyrotomy has long been established as the last-resort and potentially life-saving procedure for patients who cannot be intubated or ventilated and would otherwise face imminent death. It is essential to identify the situation and perform an emergency cricothyrotomy before the patient ends up with a cardiac arrest [1-3]. How often “cannot intubate, cannot ventilate” (CICV) situations followed by the need for a surgical airway occur depends on the location of the patient, the qualifications and experience of the health care provider, and the medical condition of the patient. The published incidence numbers of CICV situations with the use of surgical airway techniques vary from 0 to 18.5% in the studies included in Table 1 [4-19]. However, these figures should be read with caution since some figures are old and may not be representative for the present practice in the relevant services. Furthermore, the incidence will probably vary depending on how early in the applied airway management algorithm it is recommended to perform an emergency cricothyrotomy.

**Table 1:** Incidence of emergency cricothyrotomies in selected studies identified in the searches employed in this review

**Authors**	**Year**	**Country**	**Study design**	**Setting**	**Performers**	**Patient category**	**Secured airways**	**Cricothyrotomies**	
								**Number**	**Percentage**
Cook *et al *[19]	2008-2009	UK	Prospective	Hospital	Anaesthesiologists	Mix	2872600	58	0.002
Adams *et al *[4]	2005-2007	Iraq	Prospective	Prehospital	Physicians or combat medic	Trauma	293	17	5.8
Brown and Thomas [18]	1999	USA	Retrospective	Mix	Flight nurses	Mix	36	2	5.6
Germann *et al *[5]	1998-2006	USA	Prospective, single centre study	Prehospital	Flight nurses	Trauma (mainly)	369	6	1.6
Stephens *et al *[6]	1996-2006	USA	Retrospective	Hospital	Anaesthesiologists	Trauma	6088	21	0.3
Sagarin *et al *[7]	1996-2001	USA/Canada	Prospective, multicentre study	Hospital	Emergency medicine residents (mainly)	Mix	5757	50	0.9
Adnet *et al *[8]	1996-1997	France	Prospective, multicentre study	Prehospital	Emergency physicians and anaesthesiologists	Mix	691	0	0
McIntosh *et al *[9]	1995-2004	USA	Retrospective	Prehospital	Flight nurses (mainly)	Mix	712	17	2.4
Bair *et al *[10]	1995-2000	USA	Retrospective	Hospital	Emergency physicians or surgeons	Mix	201	22	10.9
Bair *et al *[10]	1995-2000	USA	Retrospective	Prehospital	Flight nurses	Mix	2259	28	1.2
Sakles *et al *[11]	1995-1996	USA	Prospective, single centre study	Hospital	Emergency medicine residents (mainly)	Mix	610	7	1.1
Fortune *et al *[12]	1991-1995	USA	Retrospective	Prehospital	EMTs	Mix	376	56	14.9
Jacobson *et al *[13]	1990-1994	USA	Retrospective	Prehospital	Paramedics	Trauma (mainly)	509	50	9.8
Nugent *et al *[14]	1987-1989	USA	Retrospective	Prehospital	Flight nurses	Trauma (mainly)	302	56	18.5
Robinson *et al *[15]	1983-1997	USA	Retrospective	Prehospital	Flight nurses (mainly)	Trauma	1589	8	0.5
Boyle *et al *[16]	1983-1988	USA	Retrospective	Mix	Flight nurses	Mix	650	69	10.6
McGill *et al *[17]	1977-1980	USA	Retrospective	Hospital	Emergency physicians or surgeons	Mix	1362	38	2.8

Traditionally, a surgical airway has been established by making an incision through the skin and the cricothyroid membrane into the tracheal lumen through which an endotracheal tube is inserted [20]. This method is not without complications, and several investigators have modified the technique [21,22]. In recent years, a number of commercial kits that include all of the necessary equipment to establish a surgical airway have reached the market. These commercial kits can be divided into two broad categories. One category depends on puncture of the cricothyroid membrane with a needle, followed by the insertion of a guidewire through the needle (Seldinger technique) [23]. A specially designed endotracheal tube included in the kit can be inserted into the trachea over the guidewire, if necessary, after the prior use of a dilator. The other category of commercial kits does not rely on the use of a guidewire; in these kits, a cutting device is employed to create a lumen in the cricothyroid membrane that is wide enough to accommodate the endotracheal tube included in the kit. Furthermore, some authors recommend that a temporary airway be established by puncturing the cricothyroid membrane with a wide-bore cannula through which the patient can be ventilated and oxygenated [20].

Because the CICV-situation is encountered infrequently, most health care providers have little if any clinical experience with the various techniques that can be employed. It is unclear which of the available methods that is most reliable and fastest to perform in the extremely time-critical CICV situations. The goal of this systematic review was to identify the current available research literature to evaluate the evidence-based information on this topic.

## Methods

### Search strategy

The electronic databases that were searched included the following: MEDLINE, EMBASE, CINAHL, Cochrane Central Register of Controlled Trials (CENTRAL), and British Nursing Index.

The full search strategies are available in the Appendix. The last update of the searches was performed on April 10, 2013. No language restriction was enforced.

The bibliographies of the included studies were also examined for other studies that could be considered for inclusion.

### Types of studies

Cricothyrotomy is an intervention that is infrequently performed; when performed, it constitutes an emergency procedure. For ethical and practical reasons, we expected few, if any, randomised clinical studies to have been conducted, as well as that most studies – independent of design – would most likely include few patients. Based upon these considerations, all prehospital, hospital and laboratory studies with living patients/participants, cadavers, manikins and animals were included.

All study designs were included.

### Types of participants

Cricothyrotomy may be necessary for patients of all ages suffering from either medical conditions or trauma. Hence, no limitations were enforced based on the age or condition (or trauma) the patient suffered.

All categories of health care providers, independent of formal training and education, could end up in a situation where a cricothyrotomy seems like a necessary intervention. Hence, no limitations were implemented in the literature searches on the background of the personnel participating in the reported studies.

### Types of interventions

The three Seldinger kits included in this review are the Arndt emergency cricothyrotomy catheter set (Arnd) (Cook, Bloomington, IL, USA), the Melker emergency cricothyrotomy (Mlkr) (Cook, Bloomington, IL, USA) and the Minitrach II (Mini) (Smiths Medical Ltd, Hythe, UK) (Table 2). Common to all kits is the performance of an initial skin incision with a scalpel before the cricothyroid membrane is punctured with a needle that is attached to a syringe. When aspiration of air confirms the tracheal position of the needle, a guidewire is inserted through the needle. Following the removal of the needle, a specially designed endotracheal tube is introduced into the tracheal lumen over the guidewire, and the guidewire is removed. An inflatable airway bag is connected to the tube, and the patient can be ventilated.

**Table 2:** Emergency cricothyrotomy methods included in this review with abbreviations used for the individual techniques

1. Kits based upon the Seldinger guidewire technique	
Arnd	Arndt emergency cricothyrotomy catheter set
Mlkr	Melker emergency cricothyrotomy set
Mini	Minitrach II
2. Kits not based upon the Seldinger guidewire technique	
Airf	Airfree
Pati	Patil’s airway
Pert	Pertrach
PCK	Portex cricothyrotomy Kit (PCK™)
QT1	QuickTrach 1 cricothyrotomy device
QT2	QuickTrach 2 cricothyrotomy device
Trqu	TracheoQuick
3. Open, surgical techniques	
Surg	Varieties of the surgical technique
Bair	The “Bair claw” device
RFST	Rapid four-step-technique
BACT	Bougie-assisted cricothyrotomy
Csci	Cricothyrotomy scissors
4. Needle techniques	
Need	Needle cricothyrotomy
Trac	Transtracheal airway catheter

The Airfree coniotomy set (Airf) (FRC Medizintechnik, Holzheim a.F., Deutschland), the Patil’s airway (Pati) (Cook, Bloomington, IL, USA), the Portex cricothyrotomy kit (PCK) (Smiths Medical Ltd, Hythe, UK), the QuickTrach1 kit (QT1) and the QuickTrach2 kit (QT2) (VBM Medizintechnik GmbH, Sulz, Germany), the TracheoQuick emergency coniotomy set (Trqu) (Teleflex Medical GmbH, Kernen, Deutschland), and the Pertrach kit (Pert) (Pulmodyne, Indianapolis, IN, USA) are the seven kits that do not rely on the Seldinger technique that have been identified in this review. In these kits, custom-made cutting devices are used to incise the cricothyroid membrane, and no guidewire is used. After the dilation of the hole, a specially designed endotracheal tube can be introduced into the tracheal lumen. Again, an inflatable airway bag is connected to the tube, and the patient can be ventilated.

Airf consists of a tube surrounding a sharp trocar. The trocar is used to incise the skin and the cricothyroid membrane, and when placed in the tracheal lumen, the trocar is withdrawn while the surrounding tube remains in place, allowing the ventilation of the patient. The QT1 and QT2 are similar to Airf, and both methods involve an artificial airway pre-loaded over a large bore needle and a direct puncture of the cricothyroid membrane. When the correct position is confirmed by aspiration of air, the needle is removed. The QT1 and QT2 can be connected to the ventilation bag with the provided flexible tubing. The Pati and Trqu are based upon the same principle, as is the Pert, but with the Pert, a splitting needle is employed.

The PCK is based on a tube-over-needle design through which the correct placement of the spring-loaded needle in the trachea is shown by a flag in the needle hub indicating tissue contact. Once the tracheal lumen has been reached, the indicator flag in the needle hub disappears, reappearing when the needle touches the posterior tracheal wall. After redirecting and advancing the device 1-2 cm caudally, the needle is removed and the cricothyrotomy tube is slid over the dilator into the tracheal lumen, and finally, the dilator is removed.

There are a number of variants of the standard surgical technique (Surg) described in the literature. Most variants make use of a scalpel, a dilator, hemostats, a tracheal hook and a tracheostomy tube [20]. The operator will make a skin incision over the cricothyroid membrane. The membrane is localised by blunt dissection before a short horizontal stab incision is made in the lower part of the membrane. The larynx is stabilised with the tracheal hook at the inferior aspect of the thyroid cartilage, and the incision in the membrane is dilated before the tracheostomy tube is advanced into the tracheal lumen.

An alternative surgical technique makes use of the Bair claw device (Bair), which can be attached to a scalpel [24]. A horizontal incision is made through the cricothyroid membrane after the palpation of the landmarks. The scalpel is withdrawn from the airway, and the device is rotated caudally before the hooks are spread with blunt dissection of the tissue. Finally, the endotracheal tube is inserted between the hooks of the device.

The rapid four-step technique (RFST) was developed from the classic surgical emergency cricothyrotomy [21]. The cricothyroid membrane is palpated before a horizontal stab incision is made through the skin and membrane with the scalpel, followed by tracheal hook traction in the caudal direction. This allows the operator to perform the procedure with minimal assistance, holding the tracheal hook with one hand while passing the endotracheal tube with the other hand. The bougie-assisted cricothyrotomy technique (BACT) is a refinement of the RFST [22]. After the incision through the skin and the cricothyroid membrane, a bougie is used to secure the tracheal lumen before the endotracheal tube is advanced into the trachea over the bougie.

The cricothyrotomy scissor (Csci) is a modified pair of scissors that are pushed with closed blades without previous incision of the skin all the way through the cricothyroid membrane into the trachea [25]. Inside the trachea, the scissors are opened, and the hole is enlarged. After closing the blades, the Csci are rotated 90 degrees; the blades are again opened; and an endotracheal tube can be passed through the resulting hole.

A needle cricothyrotomy (Need) is performed with a cannula attached to a syringe [20]. The needle is advanced through the skin and underlying tissues until the cricothyroid membrane is punctured. Aspiration of air confirms the correct intratracheal placement. The cannula is then advanced over the needle until the flanges rest on the skin and the needle is removed. An alternative to employing a wide-bore cannula intended for intravenous use is the Cook Transtracheal Jet Airway Catheter (Trac) (Cook, Bloomington, IL, USA), which can be combined with a jet ventilator. Once the tracheal catheter placement is achieved, ventilation is initiated using the manual jet ventilator connected to a high-flow oxygen source.

We have included all identified studies where two or more of the above techniques have been compared.

### Types of outcome measures

The two outcome measures studied in this review were success rate and time used to secure the airway. Most of the identified studies also had other outcome measures like complication rate and preferred technique of the performer, but success rate and time consumption were the outcome measures that were always reported.

### Study selection

MS assessed all references at the title/abstract level, while PKH, ARN and SL each independently assessed a third of the references. Disagreements were resolved through discussion between the two assessors, and when required, one of the other authors was consulted. We obtained full text articles of all studies that were not discarded on the abstract level.

### Data extraction and management

We designed a form to extract data. For eligible studies, two review authors independently extracted the information. Discrepancies were resolved through discussion.

### Assessment of the risk of bias in the included studies

Two review authors independently assessed the risk of bias for each study using the criteria outlined in the Cochrane Handbook for Systematic Reviews of Interventions [26]. Disagreements were resolved by discussion or through the involvement of a third assessor.

The risk of bias tool used for randomised controlled trials involves assessing the following five criteria:

1. Sequence generation (checking for possible selection bias)

2. Allocation concealment (checking for possible selection bias)

3. Blinding (checking for possible performance bias and detection bias)

4. Incomplete outcome data (checking for possible attrition bias through withdrawals, dropouts, protocol deviations, and use of ITT analyses where appropriate)

5. Selective reporting bias (checking if expected outcomes are reported and if there is reason to suspect publication bias)

### Measures of treatment effect

#### *Dichotomous data*

For success rate, the results are presented as summary risk ratios (RR) with 95% confidence intervals (CI).

#### *Continuous data*

The time consumption has been presented in descriptive tables with median and IQR if mentioned in the original paper. The time consumption for the procedure when the procedure failed (secure airways not obtained) was handled differently in different studies. Some studies presented the time consumption from successful placements only, excluding the failures. Other studies used a stop rule where if more than a set number of seconds were used, they were classified as failures; in these, the stop rule number of seconds were presented as the time consumption.

### Analysis and synthesis

Where we considered it appropriate to combine results from different studies, we have done so. Where we considered it inappropriate, we presented the results descriptively in tables. We carried out statistical analysis (meta-analyses) using the RevMan 5 software (RevMan 2011, http://ims.cochrane.org/revman). We expected that there would be differences among trials in both the populations and interventions, so we used random effects meta-analysis for combining data.

### Assessment of heterogeneity

The size and direction of the effects have been considered and consulted with the I^2^ and Chi-square statistics to quantify the level of heterogeneity among the trials in each analysis. Caution in the interpretation of the results is advised where substantial (I^2^ between 30 and 60%) or considerable (I^2^ between 50 and 100%) heterogeneity exists.

### Grading the quality of the evidence

The quality of the evidence for each of the critically important outcomes has been graded using the GRADE methodology (http://www.gradeworkinggroup.org) [27]. For each outcome, the quality of the evidence was assessed using the eight GRADE criteria: five considering downgrading, including study limitations, heterogeneity, directness of the evidence, precision, and reporting bias, and three considering possible upgrading, including strong effect, dose-response, and plausible confounding.

## Results

**Figure 1:**
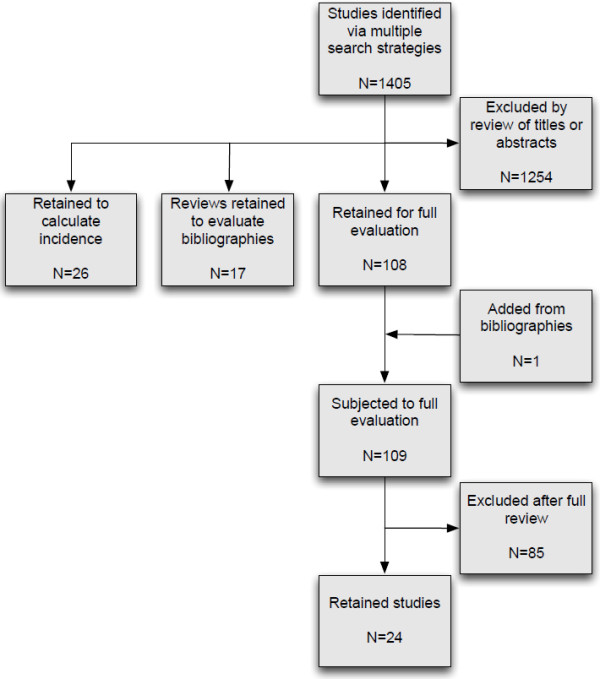
Flow chart showing the number of articles identified and excluded.

The systematic literature searches yielded 1,405 unique references, including a meta-analysis performed by Hubble and coworkers [28]. A total of 108 full text articles were retrieved, and two authors read them independently; 24 studies were included in this review (Figure 1) [21,22,24,25,29-48]. The 85 studies that were excluded in this process did not include sufficient information on comparison between two or more techniques to be included in this review. All the remaining 24 studies were prospective experimental studies with varying degrees of randomisation (Table 3). Studies involving human cadavers (ten studies), various airway simulators (eight studies), a pig laryngeal model (three studies), anaesthetised sheep (two studies) and sheep cadavers (one study) were included. The interventions were performed by students and professionals from a variety of disciplines (anaesthesiology, emergency medicine, intensive care unit physicians, medical students, paramedics). In the study involving the largest number of participants, 64 anaesthesiologists took part, while the smallest study in this respect involved two anaesthesiologists. Studies were included from the following eight countries: USA (eight studies), Germany (five studies), Austria (three studies), Australia (two studies), Ireland (two studies), United Kingdom (two studies) and one each from Canada and The Netherlands. The oldest study was published in 1993, and the most recent was published in 2012.

**Table 3:** Characteristics of the included studies

**Authors**	**Year**	**Country**	**Methods studied**	**Model**	**Participants**
Helm *et al *[48]	2012	Germany	PCK, Surg	Human cadavers	30 first year anaesthesiology residents
Helmstaedter *et al *[29]	2012	Germany	Airf, PCK, QT1, QT2, Trqu, Surg, Need	Airway model (Frova Crico-Trainer)	20 anaesthesiologists and anaesthesiology residents
Givens *et al *[30]	2011	USA	Mlkr, QT1	Manikin (Air-Man) (in a confined area)	20 emergency medicine residents
Metterlein *et al *[31]	2011	Germany	Mlkr, QT2	Cadavers of adult sheep	2 anaesthesiologists
Murphy *et al *[32]	2011	Ireland	Mlkr, PCK, QT2, Surg	Dead pig laryngeal model	20 anaesthesiologists
Hill *et al *[22]	2010	USA	RFST, BACT	Anaesthetised sheep	21 emergency medicine residents and students
Salah *et al *[33]	2010	Ireland	Mini, QT2, Surg, Need	Airway model (Bill I)	21 anaesthesiologists
Mariappa *et al *[34]	2009	Australia	Mlkr, PCK, Surg	Manikin (Portex)	4 intensive care unit physicians
Schober *et al *[25]	2009	Germany	Mlkr, QT1, Surg, Csci	Human cadavers	63 5th year medical students
Benkhadra *et al *[35]	2008	Austria	Mlkr, PCK	Human cadavers	2 anaesthesiologists
Dimitriadis and Paoloni [36]	2008	Australia	Mlkr, Mini, QT1, Surg	Airway model (locally designed)	23 emergency medicine physicians
Assmann *et al *[37]	2007	Canada	Mlkr, PCK	Manikin (Nasco cricothyrotomy simulator)	64 anaesthesiologists
Sulaiman *et al *[38]	2006	United Kingdom	Mlkr, Surg	Airway model (Bill I)	27 anaesthesiologists
Schaumann *et al *[39]	2005	Austria	Arnd, Surg	Human cadavers	20 emergency medicine physicians
Fikkers *et al *[40]	2004	The Netherlands	Mini, QT1	Dead pig laryngeal model	10 anaesthesiology residents and 10 ENT residents
Keane *et al *[41]	2004	USA	Mlkr, Surg	Dead pig laryngeal model	22 paramedics
Vadodaria *et al *[42]	2004	United Kingdom	Mlkr, Pati, QT1, Trac	Manikin (METI)	10 anaesthesiologists
Mutzbauer *et al *[43]	2003	Germany	Surg, Need	Human cadavers	18 anaesthesiology residents and 2 students
Davis *et al *[44]	2000	USA	Bair, Surg	Human cadavers	5 emergency medicine physicians
Eisenburger *et al *[45]	2000	Austria	Arnd, Surg	Human cadavers	20 intensive care unit physicians
Bair and Sakles [24]	1999	USA	Surg, Bair	Anaesthetised sheep	10 emergency medicine residents
Chan *et al *[46]	1999	USA	Mlkr, Surg	Human cadavers	15 emergency medicine attendants and residents
Holmes *et al *[21]	1998	USA	Surg, RFST	Human cadavers	28 emergency medicine interns and residents, 4 students
Johnson *et al *[47]	1993	USA	Pert, Surg	Human cadavers	44 paramedic students

**Figure 2:**
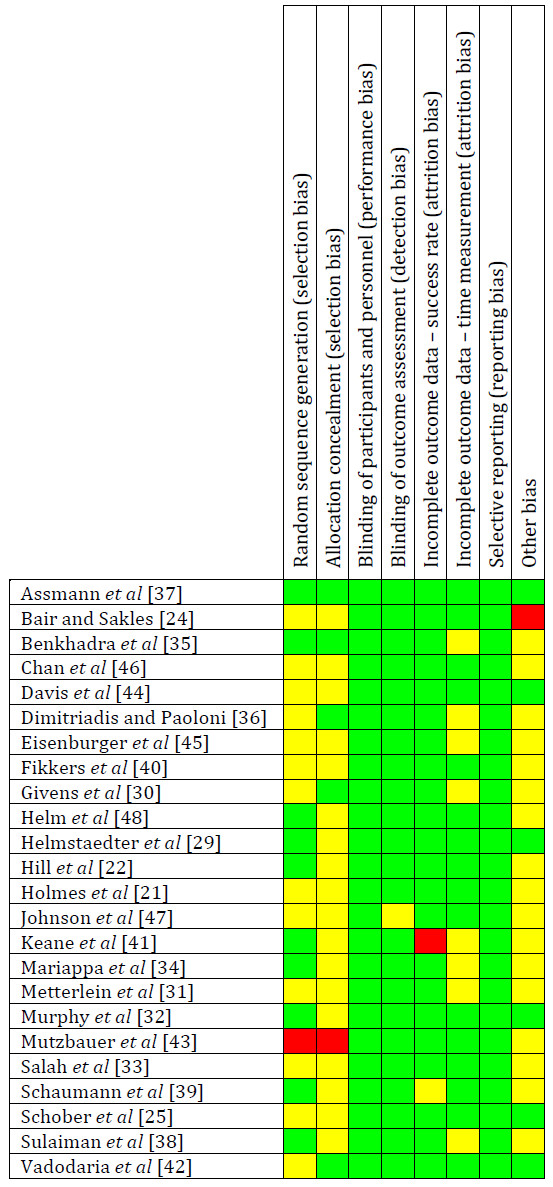
**Risk of bias analysis for all studies included in the systematic review.** Green symbols indicate low risk of bias (blinding or lack of blinding not likely to influence the results), red symbols high risk of bias, while yellow symbols indicate unclear risk of bias.

The risk of bias of the included studies is summarised in Figure 2. Methods of randomisation and allocation were poorly described in the majority of the articles. Therefore, we have concluded with an unclear risk of bias for most of the studies.

Relevant Forest plots are presented in Additional file 1 and characteristics of the included studies are summarised in Table 4.

**Table 4:** Time consumption and success rates for the various emergency cricothyrotomy methods

**Method**	**Authors**	**Time**		**Median or mean?**	**Time (sec)**	**Failure limit (sec)**	**Variation**		**Rank in individ. study (time)**	**Participants**		**Success rate (%)**
		**Start**	**Stop**				**Measure**	**Value(s) (sec)**		**Success**	**Total**	
Arnd	Schaumann *et al *[39]	NR	T3	Mean	7.9	-	SD	11	1/2	82	93	88
			T5		98.7			58.3				
			T8		108.6			59.5				
	Eisenburger *et al *[45]	NR	T3	Mean	8	-	SD	7	1/2	12	20	60
			T5		30			28				
			T8		100			46				
Mlkr	Givens *et al *[30]	T4	T6	Mean	108.5	-	Range	[59.1-219.4]	2/2	20	20	100
	Metterlein *et al *[31]	T1	T5	Median	14	180	IQR	[11-16]	2/2	8	8	100
		T5	T8		53			[52-55.8]				
	Murphy *et al *[32]	T4	T8	Median	94	300	IQR	[77-132]	3/4	20	20	100
	Mariappa *et al *[34]	T3	T8	Median	50	-	IQR	[40-56.3]	2/3	20	20	100
	Benkhadra *et al *[35]	T5	T7	Median	71	300	95% CI	[60-92]	2/2	19	20	95
	Dimitriadis and Paoloni [36]	NR	T5	Median	28	210	IQR	[25-42]	4/4	17	23	74
			T6		123			[97-210]				
			T8		126			[102-210]				
	Schober *et al *[25]	T2	T8	Median	135	-	IQR	[116-307]	4/4	10	14	71
	Assmann *et al *[37]	T2	T8	Mean	42.3	-	SD	12.5	2/2	298	320	93
	Sulaiman *et al *[38]	T4	T8	Mean	87.2	-	SD	21.6	2/2	25	27	93
	Keane *et al *[41]	T5	T6	Mean	122.7	-	SD	48.4	2/2	20	22	91
	Vadodaria *et al *[42]	NR	NR	Median	38	300	Range	[30-54]	1/4	10	10	100
	Chan *et al *[46]	T5	T6	Mean	74.7	-	95% CI	[63.2-86.2]	2/2	14	15	93
Mini	Salah *et al *[33]	NR	T8	Mean	123	40	SD	46	4/4	0	21	0
	Dimitriadis and Paoloni [36]	NR	T5	Median	21	210	IQR	[16-30]	2/4	23	23	100
			T6		41			[36-48]				
			T8		48			[40-55]				
	Fikkers *et al *[40]	T4	T5	Mean	20.8	240	SD	8.8	2/2	17	20	85
		T5	T8		149.7			44.2				
Airf	Helmstaedter *et al *[29]	T4	T7	Median	15.1	-	Range	[9.9-22.2]	4/7	20	20	100
			T8		22.8			[14.3-33.2]				
Pati	Vadodaria *et al *[42]	NR	NR	Median	123	300	Range	[74-147]	4/4	8	10	80
Pert	Johnson *et al *[47]	NR	T8	Mean	148	-	SD	96	2/2	32	44	73
PCK	Helm *et al *[48]	T3	T6	Median	104	-	Range	[51-170]	2/2	10	15	67
	Helmstaedter *et al *[29]	T4	T7	Median	29.6	-	Range	[15.9-49.1]	7/7	20	20	100
			T8		46.7			[37-67.3]				
	Murphy *et al *[32]	T4	T8	Median	181.5	300	IQR	[71-300]	4/4	12	20	60
	Mariappa *et al *[34]	T3	T8	Median	62.5	-	IQR	[41.3-150]	3/3	6	20	30
	Benkhadra *et al *[35]	T5	T7	Median	54	300	95% CI	[47-68]	1/2	16	20	80
	Assmann *et al *[37]	T2	T8	Mean	32.6	-	SD	14.9	1/2	304	320	95
QT1	Helmstaedter *et al *[29]	T4	T7	Median	13.3	-	Range	[5.5-34.2]	3/7	20	20	100
			T8		21.1			[14.5-32.4]				
	Givens *et al *[30]	T4	T6	Mean	23.9	-	Range	[8.5-63.6]	1/2	20	20	100
	Dimitriadis and Paoloni [36]	NR	T5	Median	24	210	IQR	[20-26]	2/4	23	23	100
			T6		40			[30-58]				
			T8		48			[36-75]				
	Schober *et al *[25]	T2	T8	Median	74	-	IQR	[48-145]	2/4	14	17	82
	Fikkers *et al *[40]	T4	T5	Mean	13.3	240	SD	6	1/2	19	20	95
		T5	T8		47.9			19.6				
	Vadodaria *et al *[42]	NR	NR	Median	51	300	Range	[42-73]	2/4	10	10	100
QT2	Helmstaedter *et al *[29]	T4	T7	Median	16.9	-	Range	[10.5-36.2]	5/7	20	20	100
			T8		29.9			[25-50.5]				
	Metterlein *et al *[31]	T1	T5	Median	15	180	IQR	[12-16]	1/2	5	8	63
		T5	T8		32			[29-33.5]				
	Murphy *et al *[32]	T4	T8	Median	52	300	IQR	[38-77]	1/4	19	20	95
	Salah *et al *[33]	NR	T8	Mean	72	40	SD	47	3/4	9	21	43
Trqu	Helmstaedter *et al *[29]	T4	T7	Median	13.5	-	Range	[4.6-29.5]	2/7	20	20	100
			T8		20.2			[11.4-44.7]				
Surg	Helm *et al *[48]	T3	T6	Median	95	-	Range	[43-165]	1/2	15	15	100
	Helmstaedter *et al *[29]	T4	T7	Median	23.4	-	Range	[16.2-53.2]	6/7	20	20	100
			T8		35.4			[30-61.8]				
	Murphy *et al *[32]	T4	T8	Median	59	300	IQR	[41-127]	2/4	19	29	95
	Salah *et al *[33]	NR	T8	Mean	47	40	SD	16	1/4	14	21	67
	Mariappa *et al *[34]	T3	T8	Median	47	-	IQR	[41-55]	1/3	11	20	55
	Dimitriadis and Paoloni [36]	NR	T5	Median	15	210	IQR	[10-18]	1/4	23	23	100
			T6		32			[27-60]				
			T8		34			[31-68]				
	Schober *et al *[25]	T2	T8	Median	78	-	IQR	[54-135]	3/4	17	18	94
	Sulaiman *et al *[38]	T4	T8	Mean	44.3	-	SD	12.5	1/2	23	27	85
	Schaumann *et al *[39]	NR	T3	Mean	8.2	-	SD	9.7	2/2	79	94	84
			T5		119.2			61.2				
			T8		136.6			66.3				
	Keane *et al *[41]	T5	T6	Mean	29	-	SD	14.3	1/2	20	20	100
	Mutzbauer *et al *[43]	T2	T8	Median	73	-	IQR	[60-95]	2/2	9	10	90
	Davis *et al *[44]	NR	NR	Mean	51.6	-	95% CI	[44.2-59.0]	2/2	NR	NR	100
	Eisenburger *et al *[45]	NR	T3	Mean	7	-	SD	9	2/2	14	20	70
			T5		46			37				
			T8		102			42				
	Bair and Sakles [24]	T5	T8	Median	87	-	IQR	[58-116]	2/2	NR	NR	100
	Chan *et al *[46]	T5	T6	Mean	72.8	-	95% CI	[49.8-95.7]	1/2	13	15	87
	Holmes *et al *[21]	T5	T7	Mean	133.8	-	SD	93.4	2/2	30	32	94
				Median	114		IQR	[74-154]				
	Johnson *et al *[47]	NR	T8	Mean	55	-	SD	35	1/2	38	44	86
RFST	Hill *et al *[22]	T2	T7	Median	149	300	IQR	[111-201]	2/2	8	11	73
	Holmes *et al *[21]	T5	T7	Mean	43.2	-	SD	44.6	1/2	28	32	88
				Median	32		IQR	[24-42]				
BACT	Hill *et al *[22]	T2	T7	Median	67	300	IQR	[55-82]	1/2	9	10	90
Bair	Bair and Sakles [24]	T5	T8	Median	35	-	IQR	[24-46]	1/2	NR	NR	100
	Davis *et al *[44]	NR	NR	Mean	33.3	-	95% CI	[29.8-36.8]	1/2	NR	NR	100
Csci	Schober *et al *[25]	T2	T8	Median	60	-	IQR	[42-82]	1/4	14	14	100
Need	Helmstaedter *et al *[29]	T4	T7	Median	10.6	-	Range	[5.5-23.1]	1/7	20	20	100
			T8		19.2			[10.8-27.8]				
	Salah *et al *[33]	NR	T8	Mean	56	40	SD	35	2/4	7	21	33
	Mutzbauer *et al *[43]	T2	T8	Median	25	-	IQR	[20-30]	1/2	9	10	90
Trac	Vadodaria *et al *[42]	NR	NR	Median	102	300	Range	[75-116]	3/4	10	10	100

See text for an explanation of the abbreviations for the interventions. Time points used to measure the duration of the cricothyrotomies: T1: Decides to perform the intervention; T2: Starts to palpate the neck; T3: Identifies the cricothyroid space; T4: Unwraps the device; T5: Performs the first incision or puncture; T6: Inserts the device; T7: Secures the device; T8: Performs the first ventilation; NR: Not reported in paper. Methods employed to measure variability: SD: Standard deviation; IQR: Interquartile range; CI: Confidence interval. Failure limit: The attempt to establish an emergency cricothyrotomy is defined as a failure if this time limit is exceeded (“–“indicates that no time limit is defined).

Nine groups of comparisons were included:

1. **Comparison of two kits based upon the Seldinger guidewire technique**

Arnd, Mlkr and Mini are all based upon the Seldinger guidewire technique, but only one study was identified in which two techniques based upon this principle were compared [36]. In this study comparing Mlkr and Mini, it was found that Mini had a significantly higher success rate than Mlkr.

2. **Comparison of one kit based upon the Seldinger guidewire technique with a kit that is not based upon this technique**

The Mlkr kit has been compared to Pati, PCK, QTI and QT2, respectively, and Mini has been compared with QT1 [25,30-32,34-37,40,42]. For all comparisons, no significant difference in the success rates between the two devices was detected.

3. **Comparison of one kit based upon the Seldinger guidewire technique with an open surgical technique**

Both Arnd, Mlkr and Mini have been compared to Surg [25,32,34,36,38,39,41,45],[46]. In one study, Mlkr has been compared with Csci [25]. For all comparisons, no significant difference in the success rates between the two devices was detected. In one study, Arnd was found to be a statistically faster technique than Surg. In five of the seven studies comparing Mlkr and Surg, it was shown that Surg is statistically faster. Csci was found to be significantly faster than Mlkr in the single study comparing the two devices.

4. **Comparison of one kit based upon the Seldinger guidewire technique with a needle technique**

Mlkr has been compared with Trac and Mini has been compared to Need [33,42]. In both studies, no significant difference in the success rates were identified.

5. **Comparison of two kits not based upon the Seldinger guidewire technique**

We have identified the following comparisons between two such kits: Airf vs. PCK, QT1, QT2 and Trqu, respectively; PCK vs. QT1, QT2 and Trqu, respectively; QT1 vs. QT2 and Trqu, respectively; as well as Pati vs. QT1 and QT2 vs. Trqu [29,32,42]. No significant difference in success rate was observed in any of the comparisons. In one of the studies comparing PCK and QT2, it was reported that QT2 was significantly faster than PCK.

6. **Comparison of one kit not based upon the Seldinger guidewire technique with an open, surgical technique**

A variety of the surgical technique has been compared to Airf, Pert, PCK, QT1, QT2 and Trqu [25,29,32-34,36,47,48]. In one study, Csci and QT1 has been compared [25]. For none of the comparisons, a significant difference in success was found. In the studies where Airf and Trqu, respectively, were compared to Surg, it was found that Surg was a significantly slower way to establish a surgical airway than the alternatives. The same result was found in one of the three studies where QT1 and Surg were compared. In contrast, in the single study comparing Pert and Surg, Surg was found to be the faster technique. The same result was achieved in one of the studies comparing PCK and Surg.

7. **Comparison of one kit not based upon the Seldinger guidewire technique with a needle technique**

Need has been compared to Airf, PCK, QT1, QT2 and Trqu, while Trac has been compared to Pati and QT2 [29,42]. In none of the comparisons, a significant difference in success rate was found.

8. **Comparison of two open, surgical techniques**

Varieties of Surg have been compared to Bair, RFST and Csci, respectively [21,24,25,44]. Furthermore, in one study RFST and BACT have been compared [22]. No significant difference in success rate has been reported for these comparisons. In all studies involving Surg, the alternative (that is Bair, RFST and Csci, respectively) was found to be a significantly faster option. In the single study comparing RFST and BACT, the difference in time consumption was statistically significant showing that BACT was the faster technique.

9. **Comparison of one open, surgical technique with a needle technique**

Only three studies directly compared one open surgical technique with a needle technique and no significant difference in the success rates between the two methods was detected [29,33,43].

## Discussion

This is to our knowledge the first systematic review comparing all commercial kits designed to perform emergency cricothyrotomy with surgical and needle techniques. The main result of this review is that no technique has been proven to be superior to the others, regarding success rate or time consumption. The quality of evidence is low or very low for several reasons. The studies are uniformly small, so that even though a number of studies comparing two or more techniques have been published, relatively few events have actually been analysed. When evidence is drawn from small studies, the results are uncertain and normally contain large confidence intervals. A common consequence of small trials is heterogeneity among studies, of which there are multiple examples in this review. A tendency exists toward many unique comparisons, rather than more general comparisons, and there is large variation among the roles of health care providers who perform these procedures. However, there were no studies involving surgeons. This can be interpreted that the perceived success rate with surgical techniques for experienced surgeons is so high that they do not find the commercial kits an interesting alternative even though there are no studies supporting this notion.

The studies were performed on a number of models varying from human cadavers and dead animal models to a multitude of airway models. It was difficult to make direct comparisons among studies since the primary study authors to a large degree had defined their own unique starting and stopping points in the time measurements.

Emergency cricothyrotomies are performed under stressful conditions and severe time pressure. If unsuccessful, these procedures can prove fatal or severely disabling for patients. In a laboratory setting, it is very demanding to achieve the same level of stress. Furthermore, it can be necessary to perform emergency cricothyrotomies under suboptimal conditions, such as prehospital settings, in which a lack of light, background noise and entrapped patients may add to the difficulty. Of the studies identified in this review, only one study included these types of factors that most likely affect both the success rate and the time consumption [28,30]. Furthermore, as a result of the models used, the procedures were performed without bleeding. In real life situations, bleeding will occur, thus increasing the level of difficulty of the procedure.

In the majority of the studies, there were no upper time limit that should not be exceeded for the procedure to be accepted as successful, and – with one exception – in the studies that operated with an upper limit it was between 180 and 300 seconds. An emergency cricothyrotomy will usually be performed in CICV-situation when other devices have failed and the clinical condition of the patient has already started to deteriorate. In such situations, the operator does not have 180 to 300 seconds at their disposal to perform the procedure. Salah and coworkers on the other hand chose to publish the success rate after 40 seconds, an – in our opinion – clinically much more realistic time frame, and none of the participating anaesthesiologists succeeded to establish a secure airway with Mini in this study even after five attempts [33]. In contrast, after five attempts approximately two thirds of the participants managed to establish a secure airway with the surgical technique. This study underscore the point that focusing on success rate only is insufficient; time aspects are also essential. In general, the difference in success rates may reflect varying definition of success, and operator experience, but study model probably also has an influence on outcome.

Arnd, Mlkr and Mini are the three Seldinger-based techniques included in this review, and we only identified a single study where two Seldinger-based techniques were compared. The study in which Mlkr and Mini were compared was the only study where a statistically significant difference in success rate between two devices/techniques was found. Mini was significantly faster than Mlkr, and in this study, Mini had a 100% success rate in contrast to the 74% success rate of Mlkr.

In the five different comparisons between a kit based upon the Seldinger technique and a kit not based upon this technique (Mlkr vs. Pati, Mlkr vs. PCK, Mlkr vs. QT1, Mlkr vs. QT2 and Mini vs. QT1), no statistically significant difference in the success rate was found. However, these studies were small, as were all the studies included in the review, making it necessary to interpret these results with great caution. The success rate is not the only important parameter in the context of emergency cricothyrotomies; time consumption is also critical. Individual studies tended to use their own unique combinations of starting and stopping points, making the evaluation of time comparisons difficult. Even so, it is evident that in some cases, the Seldinger-based technique was faster than the non-Seldinger technique, while in other studies, the opposite result was found. This could partly be due to different studies using different techniques and time measurements. However, when Mlkr and PCK were compared, for instance, two studies found that Mlkr was the faster technique, while two studies concluded that PCK was the faster technique. One might suspect that in those studies where anaesthesiologists performed the procedures, the Seldinger-based techniques would be faster because of the familiarity anaesthesiologists have with this technique. However, in the three comparisons of Mlkr and PCK performed by anaesthesiologists, PCK was faster than Mlkr in two of the three studies.

In four studies, a Seldinger-based technique was compared with a surgical technique (Arnd vs. Surg, Mlkr vs. Surg, Mlkr vs. Csci, Mini vs. Surg), and no statistical difference in success rate was detected. In the large majority of the comparisons, the surgical technique was faster than the Seldinger technique, even though in one study, the opposite result was found. These studies indicate that surgical techniques are generally faster than Seldinger-based kits, while both techniques have high success rates. This may indicate that surgical techniques should be favored by most healthcare providers. However, it is not unreasonable to assume that the lack of bleeding in the majority of the study models may – especially for the surgical techniques – result in artificially short procedure times and high success rates [49].

In the two studies where a Seldinger-based technique was compared with a needle technique (Mlkr vs. Trac, Mini vs. Need), the success rate was 100% for all procedures. Regarding time consumption, the results conflicted, with one study showing that the Seldinger-based technique was faster and the other showing the opposite result. This discrepancy may be explained by the fact that neither the Seldinger-based technique nor the needle technique used was the same in the two studies.

Seven different comparisons between a non-Seldinger kit and an open surgical technique were found (Airf vs. Surg, Pert vs. Surg., PCK vs. Surg, QT1 vs. Surg, QT2 vs. Surg, QT1 vs. Csci, Trqu vs. Surg). It is a weakness that five of the seven comparisons were only performed once, but this is the case for most of the currently published comparisons in this field. Conflicting results were obtained. In some instances, the non-Seldinger technique was faster, while in other instances the surgical technique was. Even in the two comparisons that were studied in more than one publication, the results conflicted, ultimately not indicating which technique was faster. The success rates were uniformly high for all devices in all studies.

There were also seven different comparisons of a non-Seldinger kit compared to a needle technique (Airf. vs. Need, Pati vs. Trac, PCK vs. Need, QT1 vs. Need, QT2 vs. Need, QT2 vs. Trac, Trqu vs. Need). All comparisons were performed only once. Each study was small, and no significant differences in success rates were detected. With the exception of the comparison between QT2 and Trac, the needle technique was found to be the faster technique, although the time differences were small and insignificant. Furthermore, it has been argued that needle techniques – in contrast to the other techniques - do not result in an airway that leads to adequate oxygenation and ventilation due to the small calibre of the artificial airway. The needle may kink and is not suitable for patient transport.

Four different comparisons between two surgical techniques were identified (Surg vs. Bair, Surg vs. RFST, Surg vs. Csci, RFST vs. BACT). All techniques had a high success rate, and no significant difference in success rate was found. However, these studies were also small, and, as was the case with all comparisons performed, any difference must be pronounced to have been detected. However, significant time differences were shown to indicate that BACT was faster than RFST, which in turn was faster than Surg. Csci was also significantly faster than Surg, but this technique has never been compared to RFST or BACT.

The field of techniques for establishing an emergency cricothyrotomy is hampered by the many very small studies performed. There is limited evidence of low and very low quality comparing these different emergency techniques for use in CICV situations. That none of the techniques produced better results than the others in these studies does not necessarily indicate that they are all equally good, and these conclusions will likely change as new evidence becomes available. This review does not justify recommending one technique over others. Success may rely on the operator’s experience and skill/training and not on the technique chosen.

## Appendix

Ovid MEDLINE(R) In-Process & Other Non-Indexed Citations and Ovid MEDLINE(R) 1946 to Present

Search strategy: 10.04.2013

1. Cricoid Cartilage/su [Surgery] 879

2. Airway Obstruction/su, th [Surgery, Therapy] 4493

3. exp Airway Management/ 84568

4. otolaryngology/ 9624

5. Neck/su [Surgery] 2893

6. ((difficult or definite or management or surgery or surgical or obstruction* or control) adj2 airway*).tw. 19262

7. (airway adj5 emergenc*).tw. 981

8. ((can?t or "can not" or cannot or diffucult* or inabilit*) adj2 (ventilat* or intubate*)).tw. 296

9. cicv.tw. 21

10. ((cricoid or neck) and (surgery or emergency or emergencies)).tw. 23509

11. (Otolaryngology or otorhinolaryngology).tw. 9401

12. or/1-11 139634

13. bougie.tw. 672

14. (single adj stab).tw. 28

15. (transtracheal adj2 ventilat*).tw. 203

16. (jet adj2 ventilat*).tw. 1276

17. or/12-16 140204

18. Tracheotomy/ 7257

19. tracheotom*.tw. 4768

20. Emergencies/ 33016

21. exp Emergency Treatment/ 88882

22. (emergency or emergencies or surgery or surgical).tw. 1258060

23. (18 or 19) and (20 or 21 or 22) 3661

24. 17 or 23 141620

25. (cricothyr* or cricotomy or cricothracheotomy or thyrocrico* or intercricothyro*).tw. 1342

26. **24 and 25 678**

27. ((quicktrach or seldinger or portex or pck or minitrach or melker) adj3 (device* or kit or technique* or set* or tube*)).tw. 774

28. (portex or cook or vbm).ti,ab. 4656

29. ((difficult or definite or management or surgery or surgical or obstruction* or control) adj2 airway*).tw. 19262

30. (cricothyr* or cricotomy or cricothracheotomy or thyrocrico* or intercricothyro*).tw. 1342

31. 27 or 28 5305

32. 29 or 30 20326

33. 31 and 32 100

34. **33 not 26 67**

Embase 1980 to 2013 Week 14

Search strategy: 10.04.2013

1. cricoid/su [Surgery] 255

2. airway obstruction/su, th [Surgery, Therapy] 3455

3. exp assisted ventilation/ 96166

4. otorhinolaryngology/ 14264

5. neck/su [Surgery]1229

6. ((difficult or definite or management or surgery or surgical or obstruction* or control) adj2 airway*).tw. 23818

7. ((can?t or "can not" or cannot or diffucult* or inabilit*) adj2 (ventilat* or intubate*)).tw. 403

8. (airway adj5 (emergency or emergencies)).tw. 1176

9. cicv.tw. 27

10. ((cricoid or neck) and (surgery or emergency or emergencies)).tw. 33129

11. (otorhinolaryngology or otolaryngology).tw. 13719

12. or/1-11 167111

13. bougie.tw. 915

14. (single adj stab).tw. 30

15. (transtracheal adj2 ventilat*).tw. 223

16. (jet adj2 ventilat*).tw. 1580

17. or/12-16 167980

18. tracheotomy/ 9157

19. tracheotom*.tw. 5525

20. emergency/ or Emergency treatment/ 45263

21. Surgery/ or ear nose throat surgery/ or emergency surgery/ or "head and neck surgery"/ 207679

22. (emergency or emergencies or surgery or surgical).tw. 1555446

23. or/18-19 11595

24. or/20-22 1633767

25. 23 and 24 3656

26. 17 or 25 169870

27. (cricothyr* or cricotomy or cricothracheotomy or thyrocrico* or intercricothyro*).tw. 1571

28. **26 and 27 783**

29. (quicktrach or seldinger or portex or pck or minitrach or melker).ti,ab,dm,dv. 2593

30. (portex or cook or vbm).ti,ab,dm,dv. 11677

31. 29 or 30 13274

32. ((difficult or definite or management or surgery or surgical or obstruction* or control) adj2 airway*).tw. 23818

33. (cricothyr* or cricotomy or cricothracheotomy or thyrocrico* or intercricothyro*).tw. 1571

34. 32 or 33 25016

35. 31 and 34 344

36. **35 not 28 273**

Cochrane Library

Date: 10.04.2013

Hits:

Clinical Trials: 34

Economic Evaluations: 2

#1 (cricothyr* or cricotomy or cricothracheotomy or thyrocrico* or intercricothyro*) (39)

#1 (quicktrach or seldinger or portex or pck or minitrach or melker) 184

#2 (portex or cook or vbm) 2997

#3 ((difficult or definite or management or surgery or surgical or obstruction* or control) NEAR/2 airway*) 2123

#4 (cricothyr* or cricotomy or cricothracheotomy or thyrocrico* or intercricothyro*) 39

#5 (( #1 OR #2 ) AND ( #3 OR #4 )) 49

#6 (#5 AND NOT #4) 45

Cinahl

Date:10.04.2013

Hits: 321

TI ( cricothyr* or cricotomy or cricothracheotomy or thyrocrico* or intercricothyro* ) or AB ( cricothyr* or cricotomy or cricothracheotomy or thyrocrico* or intercricothyro* )

(261)

(MH "Cricothyrotomy") (87)

S32 S30 and S31 (25)

S31 S28 or S29 (1102)

S30 S25 or S26 or S27 (6159)

S29 portex or cook or vbm (925)

S28 TX quicktrach or seldinger or portex or pck or minitrach or melker (227)

S27 TX difficult N2 airway or definite N2 airway or management N2 airway or surgery N2 airway or surgical N2 airway or obstruction* N2 airway or control N2 airway (5976)

S26 TI ( cricothyr* or cricotomy or cricothracheotomy or thyrocrico* or intercricothyro* ) or AB ( cricothyr* or cricotomy or cricothracheotomy or thyrocrico* or intercricothyro* ) (198)

S25 (MH "Cricothyrotomy") (72)

PubMed

Date:10.04.2013

Hits: 69 + 27

#2 Search #1 Limits: published in the last 1 year

#1 Search cricothyr* or cricotomy or cricothracheotomy or thyrocrico* or intercricothyro*

#58 Search #54 AND (#55 or #56) Limits: published in the last 1 year

#57 Search #54 AND (#55 or #56)

#56 Search cricothyr* or cricotomy or cricothracheotomy or thyrocrico* or intercricothyro*

#55 Search (difficult or definite or management or surgery or surgical or obstruction* or control) and airway

#54 Search ((quicktrach or seldinger or portex or pck or minitrach or melker) ) OR portex or cook or vbm

British Nursing Index

Date:10.04.2013

Hits: 0

(cricothyr* or cricotomy or cricothracheotomy or thyrocrico* or intercricothyro*).tw

## Abbreviations

Airf: Airfree coniotomy set; Arnd: Arndt emergency cricothyrotomy catheter set; BACT: Bougie-assisted cricothyrotomy technique; Bair: Bair claw device; CI: Confidence interval; CICV: Cannot intubate, cannot ventilate; Csci: Cricothyrotomy scissor; Mini: Minitrach II; Mlkr: Melker emergency cricothyrotomy set; Need: Needle cricothyrotomy; Pati: Patil’s airway; Pert: Pertrach kit; PCK: Portex cricothyrotomy kit; QT1: QuickTrach1 kit; QT2: QuickTrach 2 kit; RFST: Rapid four-step technique; RR: Risk ratio; Surg: Standard surgical technique; Trac: Transtracheal jet airway catheter; Trqu: TracheoQuick emergency coniotomy set.

## Competing interests

All authors declare that they have no competing interest regarding the devices and the techniques presented in this review.

## Authors’ contributions

All authors participated in all phases of the process of writing this systematic review. All authors read and approved the final manuscript.

## Supplementary Material

Additional file 1 Forest plots of all emergency cricothyrotomy comparisons performed in two or more studies. Click here for file
