# Supplementary material for: Emergency cricothyrotomy – a systematic review
Source: Scand J Trauma Resusc Emerg Med. 2013 May 31;21:43. doi: 10.1186/1757-7241-21-43 (PMC3704966; doi:10.1186/1757-7241-21-43)

a) Melker emergency cricothyrotomy set vs. Portex cricothyrotomy Kit (PCK™)


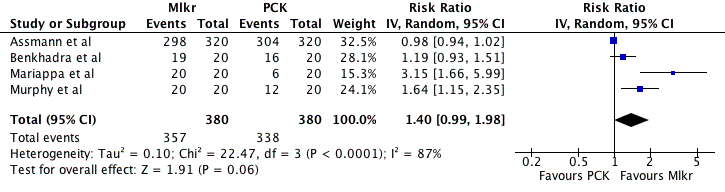


b) Melker emergency cricothyrotomy set vs. QuickTrack 1 cricothyrotomy device


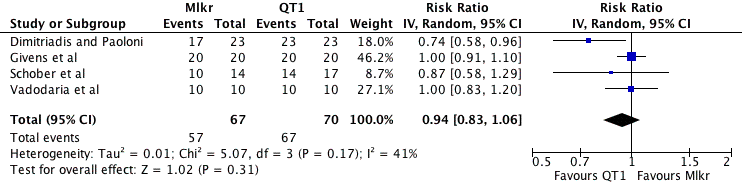


c) Melker emergency cricothyrotomy set vs. QuickTrack 2 cricothyrotomy device


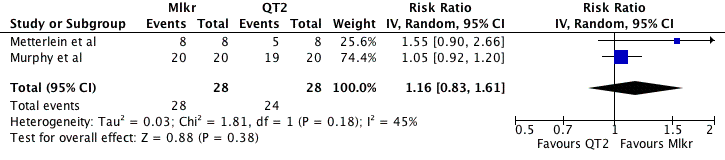


d) Minitrach II vs. QuickTrack 1 cricothyrotomy device


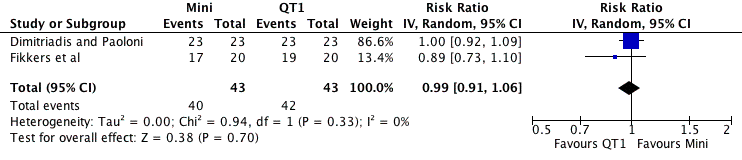


e) Arndt emergency cricothyrotomy catheter set vs. varieties of the surgical technique


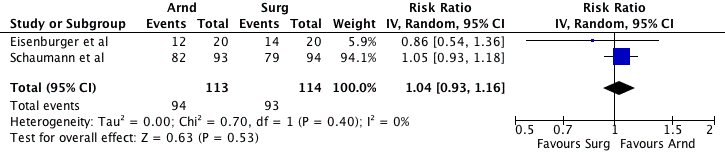


f) Melker emergency cricothyrotomy set vs. varieties of the surgical technique


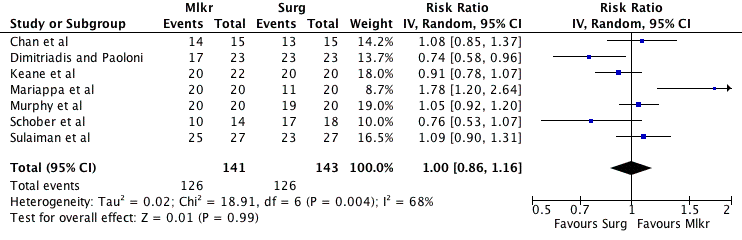


g) Minitrach II vs. varieties of the surgical technique


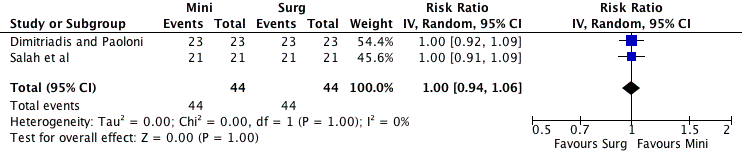


h) Portex cricothyrotomy Kit (PCK™)vs. QuickTrack 2 cricothyrotomy device


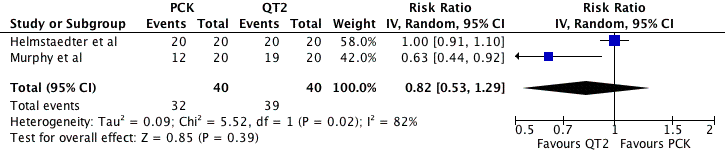


i) Portex cricothyrotomy Kit (PCK™)vs. varieties of the surgical technique


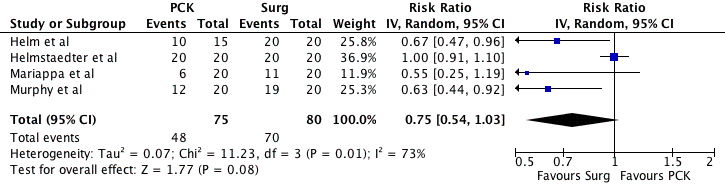


j) QuickTrack 1 cricothyrotomy device vs. varieties of the surgical technique


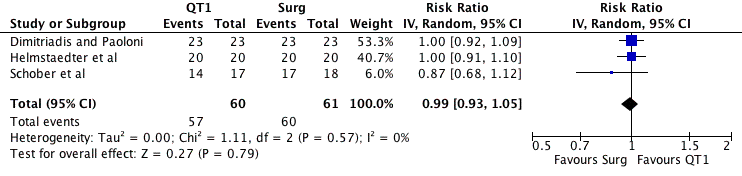


k) QuickTrack 2 cricothyrotomy device vs. varieties of the surgical technique


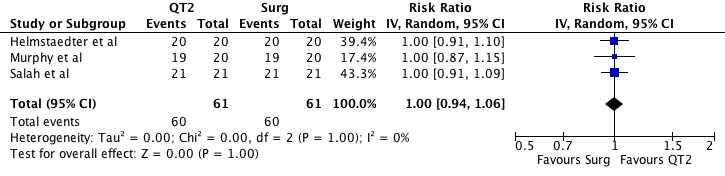


l) QuickTrack 2 cricothyrotomy device vs. needle cricothyrotomy


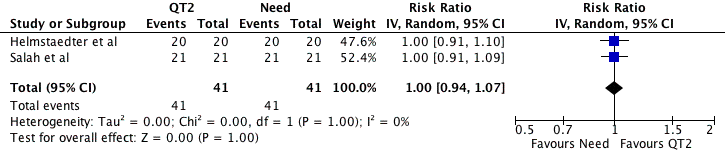


m) Varieties of the surgical technique vs. the “Bair claw” device


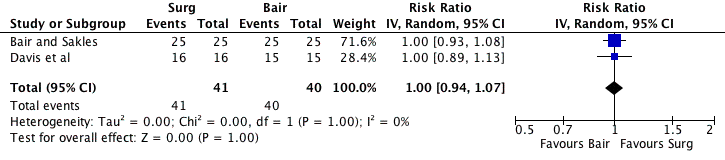


n) Varieties of the surgical technique vs. needle cricothyrotomy


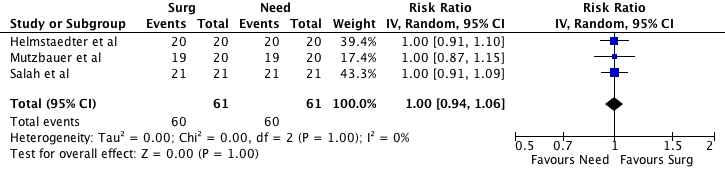

Supplement: Additional file 1 — Forest plots of all emergency cricothyrotomy comparisons performed in two or more studies. [file 1757-7241-21-43-S1.docx]
